# Tumorablative conditioning regimen for haploidentical stem cell transplantation in 102 children with hematologic malignancies: a single-center experience

**DOI:** 10.18632/oncotarget.22893

**Published:** 2017-12-04

**Authors:** Luo Rongmu, Zhang Xiaomei, Du Zhenlan, Wang Ya, Chen Wei, Si Yingjian, Gu Wenjing, Xing Guosheng, Wang Yang, Da Wanming

**Affiliations:** ^1^ Affiliated BaYi Children’s Hospital, PLA Army General Hospital, Beijing, China; ^2^ National Engineering Laboratory for Birth Defects Prevention and Control of Key Technology, Beijing, China; ^3^ Beijing Key Laboratory of Pediatric Organ Failure, Beijing, China; ^4^ Department of Hematology, Chinese PLA General Hospital, Beijing, China

**Keywords:** conditioning, haploidentical, hematopoietic stem cell transplantation, hematologic malignancy, graft-versus-host disease

## Abstract

Haploidentical hematopoietic stem cell transplantation (Haplo-HSCT) is widely carried out in China, and transplantation related complications decreased gradually with the transplant technology improving, and the overall survival(OS) increased year by year. However, relapse after transplantation is still one of the main causes of death in patients with hematological malignancy. In order to reduce the recurrence after HSCT, we set a tumorablative conditioning regimen (TAC ) regimen; the aim is as much as possible to eliminate the malignant clone to reduce the recurrence without increasing the conditioning toxicity. We retrospectively analyzed 102 cases of haplo-HSCT in our hospital from 2012 to 2017. Ninety-eight out of the 99 (99.0%) patients achieved primary engraftment. The 2-year OS and disease free survival (DFS) are 81.4% (83/102) and 77.45% (79/102). The cumulative incidence of leukemia relapse is 16.2% (16/99), Twenty-nine patients developed II-IV acute graft-versus-host disease (aGVHD) (29%) within 100 days and only nine patients have grade III-IV aGVHD (9%) in measurable 99 patients. The conditioning regimen was relatively well tolerated with limited regimen-related toxicity. The preliminary results show that TAC is safe and effective in haplo-HSCT of children with hematologic malignancies. This study will provide a clinical basis for the individualized conditioning regimen.

## INTRODUCTION

HSCT is considered an effective treatment modality for hematologic malignancies. However, post-transplant relapse is still one of the main causes of death, especially for high-risk and refractory leukemia, as recently shown in a large meta-analysis [1]. Historically, more emphasis was given to the intensity of conditioning, however, the pendulum turned more towards induction of graft-versus-leukemia (GvL) as the primary goal over the last decade. It was found that patients who suffered subsequent GvHD had a better leukemia-free survival in long term outcome and those who received intensive conditioning regimen had a higher transplant related mortality (TRM) [2]. But also enhanced GvL usually accompanied with severe GvHD, and OS had not significantly improved [3, 4]. Therefore, it is of great importance to seek a novel conditioning regimen to eliminate the leukemia as much as possible without increasing the TRM. According to the concept of tumorablative hematopoietic stem cell transplantation published previously [5, 6], in this paper, we designed a TAC combined haplo-HSCT for children with hematologic malignancies in our center.

## RESULTS

### Patients

As shown in Table 2, The patients’ characteristics are summarized in Table 2. The median age was 7.5 years (range1–18). Thirty-four patients had acute lymphocyte-B leukemia( B-ALL), 9 acute lymphocyte-T leukemia(T-ALL), 1 T and B ALL, 42 acute myeloid leukemia(AML), 8 MDS, 6 JMML, and 1 hybrid acute leukemia (HAL) and 1 Chronic eosinophilic leukemia (CEL), respectively. Overall, 29.41% of patients were not in remission at the time of transplant. 27.45% had MRD in complete remission(CR), and 43.14% had high-risk leukemia without MRD and most frequently involving poor chromosomes. All patients received G-CSF-mobilized bone marrow and PBSCs, The median follow-up interval for survived patients was 25.9 months (range 3.7–65.0).

### Engraftment

Ninety-eight out of the 99 (99.0%) patients achieved primary engraftment verified by bone marrow STR-PCR. Three patients could not be assessed died on day +14, +22 and +28 because of respiratory failure. One patient failed to achieve primary engraftment was treated with the second haplo-HSCTt from another family donor and achieved engraftment. One patient underwent secondary engraftment failure presumably because of CMV infection, and obtained complete donor chimerism after being treated by cyclophosphamide 50 mg/kg/d for two days followed by donor G-CSF-mobilized PBSC. The final engraftment of patient who can be evaluated was of 100% (99/99) (Table 1).

**Table 1:** Result in 102 patients with tumorablative preparative regimen

	Haplo-HSCT (*n* *=* 102)
Engraftment(%) (*n* ***=*** 99, 3 died before engraftment)	
Primary	99.0
Final	100
Days to ANC 500 (*n* ***=*** 101) (median, range)	15 (7–25) (*n* ***=*** 101)
Days to PLT20 000 (*n* ***=*** 101) (median, range)	16 (7–32) (*n* ***=*** 92)
aGVHD (*n* ***=*** 99) ( %) (number of patients)	
aGVHD III–IV	9.0 (9)
cGVHD II–IV	29.3 (29)
Chronic GVHD(*n* ***=*** 99)	55.5% (55)
Extensive cGVHD	10.1 (10)
Extensive cGVHD induced	4.0 (4)
Non-relapse mortality (*n* ***=*** 102) (%) ( number of patients)	
100 days	2.9 (3)
2 years	4.9 (5)
Overall	4.9 (5)
Cumulative disease progression and leukemia relapse (*n* ***=*** 99) (%) (number of patients)	
Patients (NR+PR) (*n* ***=*** 30 )	23.3 (7), 13.3 (4)
Patients (CR) (*n* ***=*** 69)	20.3 (14), 17.4 (12)
AML (except M7, CR ) (*n* ***=*** 23 )	8.7 (2), 4.3 (1)
ALL (CR ) (*n* ***=*** 40)	25 (10), 22.5 (9)
AML (NR) (*n* ***=*** 9)	22.2 (2), 22.2 (2)
Myeloid (*n* ***=*** 56)	17.9 (10), 12.5 (7)
Lymphoid (*n* ***=*** 42)	26.2 (11), 21.4 (9)
M7(*n* ***=*** 6,1 died in first month after HSCT)	33.3 (2), 33.3 (2),
MDS (*n* ***=*** 8)	0 (0), 0 (0)
JMML (*n* ***=*** 6)	33.3 (2), 33.3 (2)
All measurable patients (*n* ***=*** 99)	21.2 (21), 16.2 (16)
OS and DFS (%)	
100 days (%)	97.0, 96.0
2 year (%)	81.4, 77.5
Patients (NR+PR) (*n* ***=*** 30)	76.7, 73.3
AML (except M7,CR ) (*n* ***=*** 23 )	91.3, 87
ALL (CR ) (*n* ***=*** 41)	80.5, 78.0
AML (NR) (*n* ***=*** 9)	66.7 , 66.7
Myeloid (*n* ***=*** 57)	82.5,78.9
Lymphoid (*n* ***=*** 44)	77.3, 75.0
M7 (*n* ***=*** 7)	57.1, 57.1
MDS (*n* ***=*** 8)	100, 100
JMML (*n* ***=*** 6)	83.3, 83.3
Median follow-up survivors (months)	25.9 (range 3.7–65.0).

Abbreviations: ANC, absolute neutrophil count PLT, Platelet.

**Table 2:** Characteristics of 102 patients with hematologic malignancy and treatment

	TA-HSCT (*n =* 102)
Midian age (years) (range)	7.5 (1–18)
Gender (%)	
M	62
F	38
Donor (%)	
father	56
mother	37
sibling	7
Graft type (%)	
PB+BM	100
Disease (number of patients)	
AML	42
MDS	8
B-ALL	34
T-ALL	9
T and B ALL	1
HAL	1
JMML	6
CEL	1
Disease state (number of patients)	
NR	24
PR	6
CR-MRD (−)	44
CR-MRD (+)	28
Median number of MNCs infused (x108/kg) (range)	10.11 (3.6–41.03)
Median number of CD34+ cells infused (x106/kg) (range)	7.885 (2.23–22.74)
HLA typing (A, B, C, DR, DQ) (number of patients)	
8/10	2
7/10	8
6/10	38
5/10	54
Conditioning regimen(number of patients)	
FLAG+IDA(Mito)+VM26(VP16)+BU(TBI)/CY+ATG	88
FLAG+IDA(Mito)+VP16+BU(TBI)+ATG	11
CLAG+IDA(Mito)+VP16+BU(TBI) /CY +ATG	3

Abbreviations: F = female; FLAG: fludarabine, GCSF and cytarabine; M = male; MDS = myelodysplastic syndrome; PB = peripheral blood; IDA = Idarubicin;VM26 = teniposide;vp16 = etoposide; BU = busulfan; TBI = total body irradiation; CLAG: cladribine, GCSF and cytarabine. HAL = hybrid acute leukemia; CEL = Chronic eosinophilic leukemia, JMML = juvenile monocytic leukemia.

### GvHD

Twenty-nine patients developed II-IV aGVHD (29.3%) within 100 days in 99 measurable patients. Fifty-five (55.5%) out of 99 patients have developed chronic GvHD, only 10 (9.9%) patients were extensive manifestations. 4 of 10 extensive cGvHD caused by down-regulating immunosuppressant to induce graft-versus-leukemia (GVL) (Table 1).

### Toxicity of conditioning regimen, survival, relapse and causes of death

The conditioning regimen was well tolerated with limited regimen-related toxicity. Grade III gastrointestinal toxicity (nausea, vomiting, diarrhea, and mucositis) occurred in fifteen patients (14.7%). Reversible renal toxicity of grade III or IV never happened. One patient developed transplant related microangiopathy (grade I toxicity), Hemorrhagic cystitis (grade II-III) occurred in five patients. Liver toxicity (grade I) occurred in four patients without developing veno-occlusive disease. One patient died of diffuse alveolar hemorrhage (DAH) on day 14 after transplantation.

The primary cause of death was relapse in 14 patients and non relapse death (NRM) in 5 patients (because of unexplained pulmonary complications). Three patients died before day 100 in NRM .The 2-year OS is 81.4% (83/102) and DFS is 77.5% (79/102). The cumulative incidence of relapse rate is 16.2% (16/99), and the disease progression rate is 21.2 % (21/99).

### Infections

A total of 47 infection periods, at least grade III, developed in 36 (35.29%) patients. Most common type was pneumonia in 25 infection periods, Most common pathogenic microbes responsible for the lung infections were fungus (nine patients), bacteria (six patients), virus (four patients), pneumocystis carinii pneumonia (two patients), and unknown (four patients). Intestinal bacterial infection in 12 infection periods, viral infectious diseases in 14 infection periods, some patients have two or three infection types.

### Disease progression and relapse

The disease progression is 21.2% (21) at the end of the follow-up period, Sixteen patients (16/99) relapsed after a median of 5.75 follow-up months after transplantation (Table 1). Out of 16 relapsed patients, 9 relapsed patients occurred in ALL and 2 in JMML, 5 in AML. No recurrence in MDS and AML without MRD except M7,and only one patient developed relapse in AML with MRD except M7.

### Outcomes comparison between different leukemia

**Figure 1:**
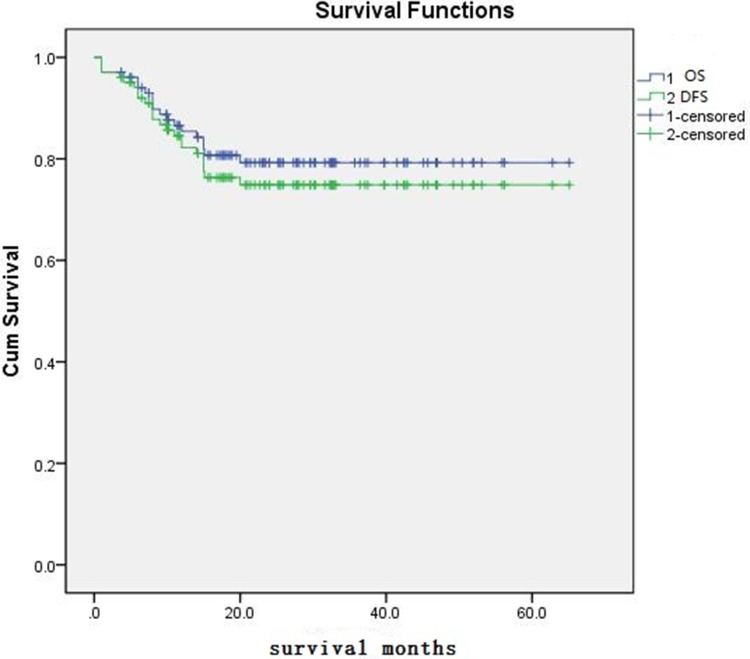
The OS and DFS of patient

As showed in Figure 1, The 2-year OS and DFS was 81.4% and 77.45%. NRM was 4.9% in two years (Table 1, Figure 1). 16 patients can not obtain CR after the last chemotherapy and 14 of MDS and JMML have not been treated efficiently before transplantation. CR was acquired in all evaluable patients after transplant. The relapse , OS and DFS of the patients without complete remission (NR and PR) before transplantation was 13.3%, 76.7%, 73.3%,respectively, which appears better than in the published studies in non-remission patients [7, 8]. The relapse , disease progression, OS and DFS in AML except M7 with CR and ALL with CR is 4.3%, 8.7%, 91.3%, 87.0%, and 22.5%, 25%, 80.5%, 78.0%, respectively(*p* = 0.124, *p* = 0.210, *p* = 0.433, *p* = 0.588,by Pearson continuity correction test), no significantly different observed. The similar also occurred between myeloid and lymphoid tumor in relapse, disease progression, OS, DFS. (12.5% vs. 21.4, 17.9% vs. 26.2, 82.5% vs. 77.3%, 78.9% vs. 75%, *p* = 0.237, *p* = 0.320, *p* = 0.804, *p* = 0.639, by Pearson chi-square test). The OS and DFS in MDS and JMML are 100%, 83.3% and 100%, 83.3%, respectively. The result indicated non-remission leukemia benefits from the study, especially for MDS, JMML, and AML-CR with MRD or not also benefit from it. however, the recurrence in M7 is 33.3% (2/6,1case died in earlier period), and OS is 57.1%, this was obviously not enough to prevent relapse in these patients.

## DISCUSSION

Allogeneic HSCT is a potentially curative treatment for hematologic malignancy, and haplo-HSCT represents an important treatment option for patients who lack a HLA-matched donor. The therapeutic effect of haplo-HSCT is mediated by both the administration of high-dose conditioning regimen and the induction of the GvL effect. The aims of this condition regimen are both suppressing host immunity to allow donor cell engraftment and eliminating the underlying malignancy. Recently, non-myeloablative (NMA) and reduced intensity conditioning (RIC) regimens were introduced to reduce transplant related toxicity and to allow in elderly and medically infirm patients [9]. Retrospective comparative trials showed that NMA/RIC is inferior in advanced disease, because of higher disease relapse [10–14]. Meanwhile, Increased dose-intensity is associated with lower relapse risk and higher NRM [10, 11]. An optional conditioning regimen should allow consistent engraftment, maximal leukemia elimination and minimal toxicity. How dose intensity of the conditioning regimen and how it may be optimized in different settings?

We designed this TAC regimen for haplo-HSCT. It attempts to eradicate malignant cells as much as possible and reduce the malignancy relapse without increasing toxicity. TAC regimen consists of high-dose chemo-radiotherapy and reduced intensity of myeloablative drugs. The scheme not only eliminates the tumor for reducing the recurrence of leukemia, but also suppresses host immunity for consistent engraftment, and without increasing the TRM. The choice of chemotherapy regimen was based on FlagI/M or ClagI/M, and combined with VP16/VM26 as the basic framework [15]. If the patient was resistant to idarubicin and mitoxantrone, we can choose other sensitive chemotherapeutic drugs. The dose of cytarabine should be adjusted in the range of 6-20 g/m^2^ according to the patient’s age and comorbidities , but more than 90% patients in the range of 10-15 g/m^2^. In addition to high-dose chemotherapy regimen, the TAC regimen also includes a reduced dose of traditional preparative drugs, such as CY, BU or TBI. We reported initial 26 cases in 2016 [15], preliminary results showed that patients with this scheme were well tolerated, no increase in TRM, the OS and DFS were above 80%. It showed strong antitumor effect to prevent recurrence. We have improved and revised the program in 2016 and the new program of AML and MDS has abandoned cyclophosphamide, because of its low eradication for myeloid malignancy and high toxicity. The suppression to host immunity of CY is replaced by fludarabine. Recently, some comparative trials showed that FLAG regimens with or without IDA /Mito produced an excellent effect in children with relapsed/refractory acute leukemia, and it could be recommended as therapeutic options prior to HSCT [16–22]. The introduction of FLAG and CLAG with IDA /Mito into the conditioning regimen is undoubtedly beneficial to the elimination of residual leukemia with acceptable toxicity, which is one of the main factors to reduce the recurrence . In addition , the strong GvL effect induced by the haploid transplant also contributed to the reduction of recurrence [23, 24].We used this program to complete a total of 102 cases, in which, 29.4% of patients did not achieve complete remission in hematology before transplantation. The 100-day TRM was 2.9%, 2 -year non-relapse mortality rate was 4.9%, OS and DFS for 2 years were 81.4% and 77.5%, respectively. The cumulative incidence rates of II-IV GVHD and severe GVHD were 29.3% and 9%, respectively, it was no increase in incidence compared with literature. 2-year cumulative relapse rate (CR = 17.4%, NR+PR = 13.3%) and a 2- year TRM of 4.9% were maintained at a relatively low level compared with previously published literature on haploid transplantation [25, 26]. The results showed that the recurrence was markedly reduced and the tolerance was good in haplo-HSCT. Comparison of different disease types, we also found that 100% DFS in 8 MDS including 7 high-risk. It is significant benefit from the TAC haplo-HSCT. 2-year OS and DFS in patient with AML-CR (except M7) even reached 91.3% and 87%, and in ALL-CR were 81.4% and 77.5%, respectively, there was no significant difference between the two groups. 6 JMML who did not receive complete hematologic remission were also obtained high survival, 2 -year OS and DFS were 83.3% and 83.3%, respectively. A retrospective analysis of 100 cases of JMML in Europe in 2005 showed that the first transplant recurrence was up to 35% [27], It showed the positive effect of TAC regimen in JMML.We also compared the differences in lymphoid and myeloid in 2- year OS, DFS by Pearson test, it showed no significant difference, and suggested that this scheme is suitable for lymphoid and myeloid neoplasms, however, it might be more beneficial trend in myeloid malignancy. In 7 cases of M7, 2 cases had recurrence and died , 1 case died from diffuse alveolar hemorrhage (DNH), 4 cases are still alive with DFS up to now., Therefore, it is very necessary to modify the conditioning regimen of M7 in order to reduce the recurrence rate .

In summary, the TAC regimen combined with haplo-HSCT showed not only lower recurrence and higher DFS , but also did not increase the risk of TRM. Although this is a small study and definite conclusion could not be drawn, it still shows that TAC regimen is very helpful to reduce the recurrence. A larger of rigorous controlled clinical study is very necessary to further confirm the efficacy and safety of the TAC regimen.

## MATERIALS AND METHODS

### Patients and study protocol

A total of 102 consecutive pediatric patients were enrolled into this retrospective study from January 2012 to January 2017 in The Department of Pediatric Hematology Oncology, Affiliated BaYi Children’s Hospital, PLA Army General Hospital, designed to assess the safety and efficacy of TAC in the treatment of haplo-HSCT in children with hematologic malignancies. The inclusion criteria were: i) less than 18 years old; ii) treated with haplo-HSCT. iii) all patients’ legal guardians signed informed consent forms. The clinical protocol and consent forms were approved by the institutional review board for human investigation at the research institution. All patients received bone marrow and peripheral blood stem cell mobilized by granulocyte colony-stimulating factor(G-CSF) (7-10 ug/kg/d) without T-cell depletion *in vitro*. Umbilical cord blood was used as the third-party in the first 4 hours before the bone marrow infusion on 0 day, The total nucleated cells (TNC) of the cord was controlled at 2.0 × 10^7^/kg [15]. The protocol of TAC includes a very effective radio-chemotherapy regimen for eradicating minimal residual disease (MRD), and a reduced intensity conventional myeloablative conditioning regimen for promoting engraftment [6] .

### HLA-typing, matching

Patient and donor were typed for alleles at HLA-A, B, C, DR, and DQB-1 by PCR amplification. The donors were parents and siblings, and the HLA-matched was 5/10 to 9/10.

### Conditioning regimen

There are three parts in TAC. The first part is effective chemotherapy regimen. It consists of FLAG or CLAG combined with a sensitive anthracycline (idarubicin,IDA, 30 mg/m^2^ divided into 3 days or mitoxantrone, Mito, 30 mg/m^2^ divided into 3 days) and etoposide(VP16) or teniposide(VM26) (300 mg/m^2^/d for 1 day). FLAG regimen contains fludarabine (200 mg/m^2^ divided into 5days), cytarabine (10.0–20.0 g/m^2^ divided into 3-5 days, the total dosages depended on the patient’s enduring of chemotherapy) and G-CSF (5ug/kg/d for 5 days) ; In advanced leukemia, CLAG regimen was preferred since 2016, which use cladribine (45 mg/m^2^ divided into 5 days) instead of fludarabine. The second part is a reduced intensity conventional myeloablative conditioning regimen, including BU (0.8-1.0 mg/kg q6h for 3 days ) or TBI (350cGy/dose for 3 consecutive days), Cy (40–50 mg/kg for 2 consecutive days ), Anti-human T lymphocyte rabbit immunoglobulin (rATG)-fresenius (20 mg/kg divided into 4 days for leukemia and myelodysplastic syndrome (MDS), 35 mg/kg divided into 5 days for juvenile monocytic leukemia (JMML) , Cyclophosphamide was abandoned after May 2016 in order to reduce conditioning toxicity in patients with AML or MDS. The detail of the conditioning regimen is showed in Table 3.

**Table 3:** Tumorablative conditioning regimen (A, B)

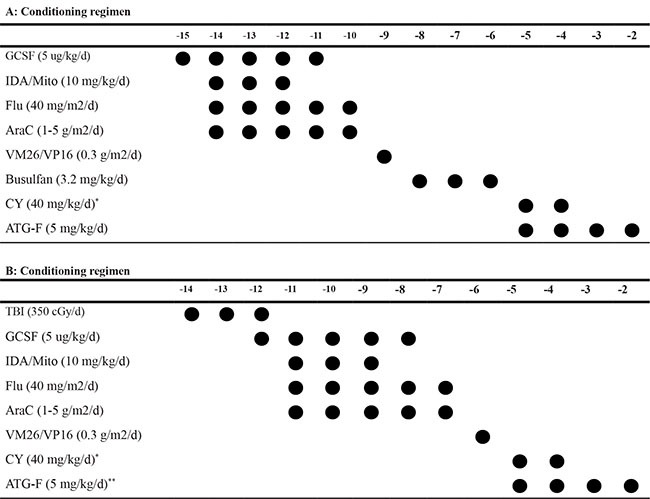

*Cyclophosphamide was abandoned after May 2016 in patients with AML, MDS and JMML

**ATG-F 20 mg/kg divided into 4 days for AL and MDS, 35 mg/kg divided into 5 days for JMML.

### Supportive care and GVHD prophylaxis

Growth factors were not routinely administered after transplant. Prophylaxis and treatment of GvHD were including tacrolimus/cyclosporine, mycophenolate mofetil (MMF), short-term mathotrexate (MTX) and ATG. MMF and tacrolimus/cyclosporine started to be administered on day −9. All patients received anti-bacterial, anti-viral and anti-fungal prophylaxis with antibiotic, ganciclovir or acyclovir, voriconazole, respectively. Pneumocystis carinii and CMV prophylaxis with gancyclovir and sulfamethoxazole, started to be administered on day -9, and acyclovir instead of ganciclovir on day +1day.

### Study assessments

The primary observation points were toxicity of conditioning regimen, engraftment of grafts, relapse of malignancy; II-IV aGVHD, TRM, and secondary observation point were chronic GvHD and infection.

### Definitions

Engraftment was defined as achieving an absolute neutrophil count (ANC) greater than 500/ul for greater than 3 consecutive days before day +30, Platelet (PLT) recovery was defined as the first day on which the PLT count was greater than 20 × 10^9^/L for 7 days not dependent on transfusing PLT. Donor derived cells detected by STR-PCR [28]. aGvHD and cGvHD were defined according to the criteria in the articles [29, 30]; Toxicity of TAC was scored using the JCO-1998 criteria [31]. The disease progression includes post-transplant relapse in any follow-up or recurring of minimal residual disease (MRD) by flow cytometry in the final follow-up in this study. Disease relapse was defined as leukemia cells greater than 5% in hematology by morphology.

### Statistical considerations

Time to event was assessed starting on the day of transplantation. OS and DFS were estimated by the method of Kaplan–Meier [32]. The incidence of disease relapse, non-relapse-mortality (NRM) and GvHD were estimated using the cumulative incidence method . Comparison of outcomes was performed on Pearson chi-square test. Statistical significance was defined as *P*-value of ≤ 0.05. Analysis was performed using SPSS 20.0 software (IBM SPSS statistics 20).
